# Supplementary material for: Are carbon emissions trading and green financial instruments synergistic? -Comprehensive quantitative research based on content analysis
Source: PLoS One. 2024 Mar 7;19(3):e0298601. doi: 10.1371/journal.pone.0298601 (PMC10919601; doi:10.1371/journal.pone.0298601)
Supplement: S1 Data — (ZIP) [file pone.0298601.s001.zip › Data/地级市Z_F工作报告.zip!/╕ú╜¿/╞╬╠∩╩╨/└┤╫╘╣1⁄2╓┌║┼í╛┬φ┐╦╩2╛▌═°í┐_2014.docx]

各位代表：

现在，我代表莆田市人民政府向大会作政府工作报告，请予审议，并请市政协各位委员和其他列席的同志提出意见。

一、2013年工作回顾

过去的一年，在中共福建省委、省政府和中共莆田市委的正确领导下，我市各级政府坚持以邓小平理论、“三个代表”重要思想、科学发展观为指导，认真学习贯彻党的十八大及十八届二中、三中全会和习近平总书记系列重要讲话精神，把握稳中求进的工作总基调，全面实施“以港兴市、产业强市”发展战略，深入开展城乡一体化综合配套改革试验，全力推进科学发展、跨越发展，顺利完成市六届人大二次会议确定的主要目标任务，向建市30周年献礼。初步统计，全市生产总值1342.86亿元，增长12.5%；财政总收入153.16亿元，增长18.7%，其中，地方级财政收入94.92亿元，增长22.6%；全社会固定资产投资1191.11亿元，增长28.0%；外贸进出口总额48.66亿美元，增长10%，其中，出口31.86亿美元，增长8.1%；实际利用外资3.02亿美元，增长18%；社会消费品零售总额428.48亿元，增长12.5%；金融机构本外币存款余额1294.78亿元、增长20%，贷款余额1094.83亿元、增长19.9%；城镇居民人均可支配收入27233元，增长10.3%；农民人均纯收入11600元，增长12.5%；居民消费价格指数上涨2.5%；城镇登记失业率2.0%；人口自然增长率7.13‰；完成节能减排年度目标。

一年来的主要工作和成效是：

（一）产业转型升级加快。全力以赴稳增长、调结构、促转型，出台促进工业、现代农业、现代渔业、现代物流业、建筑业、电子商务、木材加工区发展等政策措施，产业实力不断壮大。第一产业增加值114.58亿元、增长3.1%，新增市级以上农业产业化龙头企业19 家，发展设施农业6000亩；莆田荔枝、仙游金沙薏米列入国家地理标志商标名录，莆田鲍鱼、莆田花蛤入选全省十大特色渔业品牌。第二产业增加值 783.46亿元、增长14.3 %，规模以上工业增加值603.95亿元、增长15.3%，赛得利差别化纤维、海峡纺织城、仙游抽水蓄能电站等项目建成投产，新增规模工业企业126家；鞋服等十大产业集群发展水平提升，产值1870.52亿元，增长15.6%；新一代电子信息技术等五大战略性新兴产业加快发展，实现产值285.10亿元，增长23.5%；高新技术产业产值占规模以上工业产值26%。第三产业增加值444.82亿元，增长11.5%，商品销售总额1153.13亿元，净增限上商贸企业122家，总数达598家；文化产业增加值112.80亿元，占地区生产总值8.4%；我市列入国家电子商务示范城市和智慧城市试点，网商发展指数位列全国百强城市，快递业务量排名全国第41名。省科技创新型城市试点全面推进，专利授权量1705件，增长42.4%，荣膺全国科技进步先进城市“五连冠”。

（二）重点项目有力突破。市重点项目完成投资983亿元，“五大战役”项目完成投资1145亿元，央企、民企、外企“三维”项目完成投资589亿元。30个重点攻坚项目有力推进，交通、水利、能源等项目加快建设，向莆铁路、湄洲湾港口铁路东吴支线、沈海复线高速公路莆田段竣工通车，莆田机场前期工作取得新突破。新增铁路运营里程 45公里、高速公路通车里程115公里、风力发电装机容量13万千瓦，港口泊位吞吐能力建成128万吨、在建4500万吨。金鹰林浆纸一体化项目获得国家核准，罗屿40万吨铁矿石码头加快建设，兴化湾港区陆域形成和石门澳产业园基础设施全面推进，临港“金木水火土”五行基地构架加快形成，发展基础进一步夯实。

（三）宜居港城品质提升。城乡规划体系不断完善，城镇空间布局优化，城市功能和品位持续提升，中心城区辐射带动和综合服务能力增强。供水、供气、供电、道路交通和污水垃圾处理等市政基础设施进一步完善，改造荔涵大道、新建壶公路等城市道路50公里，改造提升市区12条主要街道，新建污水管网148公里、供水管网11.6公里、燃气管网68.3公里，城市供水普及率达98%，污水处理率达83.5%，生活垃圾无害化处理率达99.1%。提升园林绿化水平，完成造林绿化19.5万亩，新增公园260公顷，新建绿地96 公顷，推进绶溪公园和荔枝林绿道连线成片，城市绿化覆盖率、全市森林覆盖率分别提高1.5个和0.78个百分点，获得“全国绿化模范城市”称号，木兰陂获评“国家级水利风景区”，九龙谷成功创建国家4A级旅游景区。深入开展道路交通、内河、“两违”专项整治行动，新增公交车240辆、出租车100辆、公共停车位4959个，治理城区内河40公里，制止、拆除违法建筑56.3万平方米。推进节能减排，强化工业园区和养殖业污染治理，拆除关闭畜禽养殖场261万平方米，空气质量居全省第一位。房地产保持平稳健康发展，普通商品住房供应加大；保障性安居工程新开工10312套、竣工5984套，超额完成省下达任务；改造棚户区（危旧房）3100套，造福工程搬迁16400人；安置房新开工175.7 万平方米，竣工92.4万平方米；新增归集住房公积金14.2亿元，增长19.1%。清理供而未用土地8200亩，积极解决安置房历史遗留办证问题。编制城乡一体化发展规划，9个省、市小城镇综合改革试点顺利推进，16个“幸福家园”试点村实施142个项目、完成投资11亿元，有序推进农村集体土地房屋产权确权发证，城乡居民养老保险率先一体化，形成一批土地综合治理、产业支撑、乡村旅游等不同类型的典型示范，4个村列入全国“美丽乡村”试点村。

（四）改革开放积极推进。重点领域改革不断深化，重组整合市属国有企业，“八大集团”资产总额达366亿元；城市综合管理列入全省试点，下放城市管理行政执法权，进一步理顺城市管理体制；创新海域海岛管理，成立全省首家市级海域海岛储备中心；持续深化林权改革，林权发证率达98%；实施国家基本药物制度，开展村卫生所新农合普通门诊统筹试点和药品零差率销售改革试点工作。口岸开放进展顺利，通关环境不断优化。木材物流集散基地进一步完善，木材进口量48.13万立方米，增长83%。积极参加“9·8”投洽会、侨洽会、港澳经贸周等招商活动，签约项目63个，总投资51.7亿美元；成功举办第15届妈祖文化旅游节、第8届海峡艺博会、首届仙游红木博览会等；对台交流合作进一步深化，外事外侨工作继续加强，与马来西亚诗巫市等国际友好城市往来更加密切；莆抚、福莆宁平、闽东北五地市协作深入推进。

（五）民生改善落到实处。市财政安排民生支出103.4亿元，占财政支出71.8 %，市级财政超收资金全部用于民生和社会事业薄弱环节建设，16件为民办实事全面完成。就业和社会保障水平提高，新增城镇就业 2.22万人、农村劳动力转移就业3.5万人，最低工资标准提高11.7 %，企业退休人员养老金提高20.9%；独生子女伤残死亡家庭养老补助金每月从400元提高到1000元，城乡低保标准分别提高32%和60%，发放城乡低保、五保救助金2.2亿元，惠及10万困难群众；发放重度残疾人生活补助金661.9万元，惠及3.1万名贫困残疾人，市社会福利中心、市慈康医院投入使用；实现基本医疗保障政策城乡统筹并轨，新增参合人数7.9万人，参合率达99.9%；实施农村饮水工程，提升43.8万群众饮水安全水平。

（六）社会事业协调发展。新建、改建、扩建幼儿园和中小学校舍243幢，新增中小学学位9513个，标准化学校建设完成率达98.9%，莆田学院仙游校区、莆田一中妈祖城校区投入使用，“百校帮百校”活动深入开展，“班班通”列为全国典型示范案例。市儿童医院等4所医院投入使用，新增床位1596张，市第一医院、莆田学院附属医院医疗集团发挥效应，优质医疗资源扩展到南日岛、湄洲岛等基层医疗机构，公共卫生服务实现城乡全覆盖。人口计生工作完成省下达任务，“两非”现象有效遏制，稳定低生育水平，出生人口素质不断提高。文化惠民工程深入实施，“三馆一宫”加快建设，有线广播、农家书屋实现村级全覆盖；成功承办第五届世界龙狮锦标赛等赛事，鲤声剧团莆仙戏《目连救母》获得第六届巴黎中国戏曲节最佳传统剧目奖，电影下乡放映超万场；举办首届中国（莆田）书画艺术节，成立福建省油画行业协会；《莆田市志》（1995年-2005年）出版发行；新增4处全国重点文物保护单位。社会治理能力提升，治安视频监控系统实现重要部位和场所全覆盖，社会治安明显好转；加强质量标准和产销监管，食品药品安全形势总体稳定；落实安全生产责任，开展重点整治百日行动，事故死亡人数下降；畅通信访渠道，推进积案化解，依法解决群众合理诉求；有效应对 “苏力”等强台风暴雨袭击及仙游地震，突发公共危机事件应对能力提高。国防、人防和后备力量建设有新发展，军政军民关系和谐融洽，荣膺新一届“福建省双拥模范城（县）”称号。工青妇、老龄、统计、人事、编制、保密、档案等工作得到加强。对口援助、老区、贸促、新闻广电、民族宗教、红十字会、社会科学等工作取得新进步。

（七）行政效能有效提高。严格落实中央“八项规定”，切实解决“四风”方面存在的突出问题。自觉接受人大和政协的监督，办理人大代表建议203件、政协委员提案260件。推进“六五”普法工作，开展行政复议委员会试点工作，受理行政复议案件155件，办结率100%。开展“政风行风人民评”媒体直播活动，提升“12345”政务服务水平，受理群众诉求17195件，办结满意率90.9%。推进行政服务中心标准化建设，开展行政审批 “四减五增”活动，市级审批事项减少115项，即办事项比率提高121.3%。坚持“四下基层”，改进文风会风，厉行勤俭节约，清理办公用房，加强公务车辆管理，规范公务接待，停建楼堂馆所，降低行政成本。深化机关效能建设，效能问责578人次，有力整治“庸懒散奢”现象。深化行政监察和审计监督，惩治和预防腐败体系建设取得新进展。

各位代表，成绩来之不易，这是中共福建省委、省政府和中共莆田市委正确领导的结果，是全市人民齐心协力、团结奋进和各方面大力支持的结果。在此，我代表莆田市人民政府，向全市人民，向人大代表、政协委员、各民主党派、工商联、各人民团体、无党派人士、离退休老同志和社会各界人士，向中央、省直各部门及驻莆机构、驻莆部队、武警官兵、公安民警，向关心支持莆田发展的香港特别行政区同胞、澳门特别行政区同胞、台湾同胞、海外侨胞和国际友人，表示衷心的感谢！

在看到成绩的同时，我们也清醒地认识到发展还面临不少困难和问题，主要是：经济总量小，龙头企业带动作用不强，重大项目落地难、启动慢，投资增长和发展后劲不足；城乡规划建设管理水平不高，生态环境保护和节能减排压力较大；就业、就学和就医等民生保障能力与人民群众的期待仍有较大差距，公共服务体系有待完善；征地拆迁、安全生产、社会治安、食品药品安全等方面还存在不少问题，社会治理面临新的挑战；一些政府工作人员思想不解放、作风不扎实、执行力不强的问题仍然突出，极少数人甚至存在不作为、乱作为和消极腐败现象。对于这些问题，我们要高度重视、认真面对，采取更加积极、更加有力的措施切实加以解决。

二、2014年工作安排

今年政府工作的总体要求是：深入贯彻党的十八大及十八届二中、三中全会和习近平总书记系列重要讲话精神，全面落实中央和全省经济工作会议、城镇化工作会议、农村工作会议要求，按照市委的部署，坚持稳中求进、改革创新，以全面深化改革为引领，持续城市建设“一二三四五”、产业发展“金木水火土” 的工作思路，实施深化“五大战役”重点行动计划，着力提高经济发展的质量和效益，着力推进城乡一体化，着力保障和改善民生，着力保护生态环境，着力转变政府职能，努力走出一条立足莆田市情、富有莆田特色的依港口、靠文化、重科技、创模式、促转型的质量型、生态型、内涵式的发展道路，加快建设“人兴业茂、清风明月”的宜居港城。

经济社会发展的主要预期目标是：全市生产总值增长12.5%，预期1550亿元，其中，第一、二、三产业增加值分别增长3.5%、13.5%、13%；财政总收入增长17.5%，预期180亿元，其中，地方级收入增长18%，预期112亿元；全社会固定资产投资增长22%，预期1450亿元；社会消费品零售总额增长14%，预期488亿元；外贸进出口总额增长8%，预期52.5亿美元，其中，出口总额增长7%，预期34.1亿美元；实际利用外资增长13%，预期3.4亿美元；城镇居民人均可支配收入增长11%，预期30230元；农民人均纯收入增长12.5%，预期13050元；居民消费价格指数上涨3%左右；城镇登记失业率控制在3%以内；人口自然增长率控制在 7.5‰以内；完成单位生产总值能耗、化学需氧量、二氧化硫、氨氮、氮氧化物等年度节能减排任务。

实现上述目标，必须做好以下工作：

（一）推动临港产业集群加快发展

培育发展临港产业。围绕抓龙头、铸链条、建集群，培育一批具有核心竞争力的临港龙头企业，加快临港“五行”基地建设。促进鞍钢冷轧、赛得利差别化纤维一、二期、海峡纺织等项目达产；确保国投配煤基地、中海福建应急抢维修中心、赛博思钢构等项目竣工；加快建设LNG5、6号储罐、湄洲湾第二发电厂、PA6（聚酰胺切片）、蓝海新材料、华峰工贸等项目；开工建设金鹰林浆纸一体化、赛得利差别化纤维三期、丁基橡胶、雪津英博啤酒扩产等项目；有效突破莆田核电、燃气电厂二期、阿斯创矿业、神华煤电、中锦化工、庆华甲醇、泰盛纸业、青山浆粕等项目前期工作。继续实施进口木材补贴政策，扶持木材加工龙头企业，创建国际硬木交易中心，提升木材加工区集聚能力。

完善港口集疏运体系。大力建设莆田港湄洲湾、兴化湾港口群，续建秀屿、莆头、罗屿、东吴作业区等18个深水泊位，建成涵江、宫下、黄瓜、小日陆岛交通码头，抓好枫亭码头前期工作；续建湄洲湾三期航道工程，新建兴化湾港区一期航道工程。建成国投煤炭码头专用线和福厦铁路仙游客运站，加快建设湄洲湾港口铁路罗屿支线和秀屿支线；全力推进兴化湾港区、三江口、罗屿、东吴、国投配煤基地等片区疏港公路建设；继续做好莆田机场前期工作。加快建设海西国际商贸物流园、黄石商贸物流园、东吴临港物流园、海西LNG物流配送中心、东南沥青物流产业园、莆头木材集散基地等临港物流园区；发挥向莆铁路带动效应，大力拓展港口腹地，落实支持物流发展优惠政策，开工建设普洛斯物流中心，引进更多国际国内知名物流企业，推动港铁联运，促进临港物流发展。

优化港口发展环境。成立港口发展促进委员会，鼓励各类投资主体参与港口开发经营，筹建港口物流交易中心和保税仓储功能区。积极推进东吴港口全面开放，加快建设东吴口岸联检设施，建设电子口岸，提高通关效率。完善港口应急救援机制，修建海上指挥中心、海上搜救中心和海上卫星导航系统。改造搬迁罗屿半岛，拓展临港用地。

（二）增强产业实力和竞争力

促进制造业提质增效。滚动实施“双百工程”， 抓好存量提升和增量优选，促进工业化与信息化深度融合，力争工业增加值净增100亿元，技改投资突破200亿元，亿元以上企业突破500家。培育壮大新能源、新材料、节能环保等新兴产业，力争产值增速超过25%。建立小微企业转规升级培育库，重点扶持50个高成长性企业发展，净增规模企业70家。实施品牌战略，助推企业上市。抓好第三次全国经济普查工作。

做大做强重点产业。把培育和壮大龙头骨干企业作为主攻方向，引导小微企业围绕龙头企业协作配套，逐步形成龙头引领、链条延伸、集群共进的格局。纺织鞋服产业加快转型升级步伐，开发使用差别化纤维、高性能鞋材，推动“机器换人”、“技术换工”等信息化技术应用，建设全省重要的纺织服装生产基地和全国性的鞋服专业市场；食品加工产业以生物技术和工程技术为支撑，以海洋食品制造为重点，提升精细加工、产品包装、市场开发水平；医疗器械产业大力发展药物制剂和医疗器械，逐步构建新药发展基地、医药医疗器械生产和交易基地；电子信息产业加大技术创新研发力度，促进钟表、计算器、数字家电、光电行业高端化发展；装备制造业加快建设兴化湾港区机械装备园，培育金属制品、汽车摩托车配件、数控机械、石材机械、木制品加工机械等行业龙头企业，提高市场竞争力;化工产业积极发展橡胶制品、塑料制品、涂料和鞋用胶水等终端产品，重点发展精细化工新材料，延伸湄洲湾石化产业链;建筑业打造一批特色专业企业，支持总承包企业加快发展。

提速发展服务业。把第三产业作为推动经济转型升级的重要抓手，不断拓宽空间、优化结构、提高比重。提升莆田网军，打造电商名城，建设垂直细分行业、跨境电子商务等特色平台，建立新型结算中心，培育限上电商企业，加快安福电子商务城转型升级，提高企业电子商务应用水平，促进电子商务、快递业融合发展和产业集聚，创建国家电子商务示范城市。培育引进财务和资产管理公司、结算中心、评估、咨询、金融担保、传媒、代理等中介机构，大力发展医疗服务、养老休闲、养生保健等健康服务业。加快建设智慧城市，发展软件及信息技术服务外包，促进信息消费。办好各种体育赛事、演艺和会展，拉动住宿、餐饮业消费。加强金融对实体经济的服务，鼓励更多金融机构入驻莆田、增设分支机构，发展村镇银行，推进市农商行增资扩股；创新金融产品和服务模式，创设更多小额贷款公司，完善“一个行业一个政策一个协会一个资金池”机制；加强民间融资监管，打击非法集资。保障土地合理供应，加快普通商品住房建设，发展楼宇经济、总部经济，促进房地产业持续健康发展。

推进旅游业跨越发展。以“南湄洲、北九鲤”为重点，全力支持湄洲岛建设朝圣岛、旅游岛、生态岛，完善基础设施，美化旅游景观，配套娱住功能，争创国家5A级旅游景区；加快建设通往景区干道，改造提升环山区道路，联接九鲤湖、九龙谷、菜溪岩、九座寺、仙水洋、永兴岩等重要景区。提升旅游产品品质，规划建设滨海景观带，发展红色旅游、生态旅游、乡村旅游和工业旅游等业态。争取列入赴台“个人游”试点城市。

强化科技创新。加强省创新型城市试点工作，实施“十大科技创新工程”。继续扶持科技“小巨人”企业，发展高新技术产业，支持莆田高新区加快发展。加快建设海西研究院莆田中心，深化产学研合作，加强知识产权运用和保护，积极推进科技成果、专利技术产业化。实施高端人才集聚计划，培养和引进一批科技领军人才、高技能人才、复合型人才和创新团队。

推动外贸转型升级。落实“助保贷”等政策，鼓励鞋服等传统行业加强研发设计，创立自主品牌，促进差异化发展，提高产品附加值和竞争力。扩大先进技术设备、关键零部件、天然气、木材等进口。实行引资引技引智相结合，培养和引进国际化商务人才。加强莆港澳旅游、文化、科技创新等交流合作。落实省先行对台开放15项试点政策，推动莆台货运直航，组建莆台农业合作联盟。拓展与新加坡、印尼、马来西亚等莆商集聚区的合作空间。

（三）提升农业现代化水平

保障粮食安全。健全“三农”投入稳定增长机制，完善农业补贴政策，发展新型农村合作金融组织，加大农业保险支持力度。推广良种作物60万亩，稳定粮食生产。创新经营体系，推动土地适度规模经营，提高农业技术装备水平，巩固农业基础地位。整合中央和地方粮库布局，建设仙游省级粮库，筹建兴化湾粮食仓储加工物流园区。加强副食品生产基地建设，推进农超对接，确保市场供应量足价稳。建立食品原产地可追溯制度和质量标识制度，治理“餐桌污染”，保障食品安全。

大力发展设施农业。推广“山区学利农、海洋建牧场”模式，以农副产品加工现代化带动农业规模化，打造全国设施农业批发后援基地。加快建设国家级现代农业示范园、仙游台湾农民创业园、福建农民创业基地和中国（仙游）精致农业产业园，大力发展设施蔬菜、畜禽养殖、水果、茶叶、花卉、食用菌和林下经济，新增大棚蔬菜4000亩、楼房养猪5万头。加强以设施农业为重点的技术研发，推广水肥一体化等机械装备，推进无土基质栽培等现代技术应用。完善水利基础设施，支持农田水利重点县建设，推进海堤、水库、水闸除险加固，创新农田水利最后1公里建设管护机制，建设高标准农田。

加快建设海洋牧场。实施“风行海西、鲍打天下”发展战略，立体开发海上风电与海洋牧场，打造南日海上风电示范基地；实施鲍鱼、海参、海带、紫菜、牡蛎、花蛤、对虾等优势品种的种业工程，鼓励发展远洋捕捞业，加快发展现代海洋渔业和海洋服务业，建设埭头半岛渔业加工贸易区、中海源海洋高新技术产业园、后海现代渔业产业园，打造南日海洋经济综合开发试验区。推进海域收储试点，建立海域使用储备制度。推进南日十八列岛、平海湾滨海综合开发，建设中国（南日岛）垂钓基地。

着力帮扶老少边岛相对落后地区。大力扶持50个老少边岛相对落后村，优先安排水利、饮水和公路等项目，促进电网升级改造和农村信息化建设，继续实施造福工程、土地综合治理、环境卫生整治，支持教育、卫生、文化等社会事业建设。落实专项资金，扶持“一村一品一业”，增强贫困村自我发展能力。

（四）积极稳妥推进城乡一体化

有序推进农业转移人口市民化。创新人口服务和管理，改革城乡一体化户籍管理制度，实施居住证制度，推进“村改居”，建立转户进城农民权益协同保护机制，引导和鼓励农业转移人口优先向建制镇转移。推动基本公共服务向常住人口覆盖，将符合条件的流动人口纳入城镇住房和社会保障体系，促进进城农民在地域转移、职业转换的同时，实现身份和生活方式的转变。推进产城融合，培育发展各具特色的城市产业体系，提高服务业比重，增强城市产业和人口的承载能力。

优化城镇空间布局和形态。大力推进新型城镇化，做强做美中心城区和仙游城关，逐步构建“现代骨、传统魂、自然衣”的新型城乡格局。按照“一溪两岸”联动发展思路，动工建设八二一路跨木兰溪大桥，加快建设木兰大道一期，全面启动妈祖城核心区、莆田火车站片区建设，加快发展滨海新城区，推动城区跨木兰溪发展。按照城涵同城化发展思路，加快福厦路、荔涵大道改造提升，动工建设白塘路，改造白塘湖片区，综合整治城涵河道，开发建设萩芦山庄、大学城片区，促进城涵路通、船通、绿道通，增强中心城区辐射效应。

完善城市功能。实施交通、能源、市政、水利、信息、环保六项提升工程，深入推进“五千工程”。按照“一次性设计到位、五年内不破路”要求，统筹规划建设市政道路、桥梁、停车场、人行过街通道及地下管网，继续推进城区主要街道改造提升工程，开展杆线整治、推进共杆共线。按照 “五年任务三年完成”的要求，续建湄渝高速莆田段，全面启动新一轮“三纵一横三联”国省干线建设，重点建设联十一线、联一线、联二线，改造农村公路100公里，实施农村公路安保工程400公里。加快推进防洪防潮、水源保护、引调水、供排水、流域治理等重大水利项目，两年完成木兰溪整治工程，整合现有自来水厂、扩建第二水厂、建设第三水厂和妈祖城水厂，完成金钟水利枢纽工程和金莆供水工程，加快推进秋芦溪流域的乌溪水库及供水系统配套工程，开展宁海闸、河库联通、供水管网互联互通前期工作。完善高新区、经济开发区、工业园区、物流园区等配套基础设施。

改善城乡人居环境。实施宜居环境建设行动计划，延伸拓展“点线面”攻坚，把“点”从城乡社区向棚户区、城镇“三边三节点”和美丽乡村拓展，“线”从公路铁路沿线、绿道向市政道路管线拓展，“面”从小流域向木兰溪流域和旅游景区拓展，构建景观走廊。沿袭城乡自然生态和历史文脉，保护历史建筑、历史文化街区。大力实施城市绿化美化花化彩化，加快建设城市主要出入口门户景观、带状绿地、立体绿化、屋顶绿化，建设一批园林植物园、湿地公园、郊野公园、森林公园，促进绿道网连线成片，打造莆田“慢生活”。 提升主要节点夜景工程，推进路灯照明节能。

加强城镇管理。推进“四规合一”， 加强控制性详细规划和“三区四线”管控，用好乡村规划师。稳步推进城市综合管理试点工作，提升城市治理能力，提高市政设施完好率，创建一批示范街道、社区。深化道路交通、内河、“两违”三项整治，加强电动自行车、摩托车管理，提升交通组织水平和公交分担率，改善城市交通秩序；继续整治城区内河、南北洋水系，打造“水清、河畅、岸绿、景美”的河道景观；加大“两违”整治力度，坚持属地管理、部门联动，依法依规、疏堵结合，做到“零容忍、零增长”，规范城乡建设用地行为。发展绿色建筑，加强建筑市场和工程质量管理。

加快试点村（镇）建设。推动城市基础设施、公共服务设施向农村辐射延伸，逐步实现城乡道路交通、供水、供电、燃气、绿化、信息通讯、垃圾污水处理等设施联网对接，形成城乡一体化的建设和管理体系。抓好9个省市小城镇试点镇、第一批16个“幸福家园”试点村和第二批3个镇、5个片（含14个村）、16个村 “幸福家园”试点村（镇）建设，促进农民就地安居创业，打造更多不同类型、不同层次的典型示范，创新“就地城镇化”路径；在试点村开展集体经营性建设用地收储改革试点，建设统一的农村产权交易平台，有序推进农村集体土地确权发证，慎重稳妥推进农民住房财产权抵押、担保、转让。

（五）全力打造文化名城

提升莆田文化内涵。培育和践行社会主义核心价值观，着力提高市民文明素质，深化群众性精神文明创建活动，把传统美德和时代新风渗透到乡规民约、祖训家教、庙会节庆等民俗文化载体中，弘扬清风正气，树立学习典型，营造良好社会风尚。开展地域文化研究，扩大对台文化交流，拓展妈祖文化、莆商文化内涵，挖掘和凝炼历史文化名人的优秀品质，铸造新时期莆田人文精神。保护传承优秀文化遗产，加强文物保护和利用，推进莆仙戏申遗工作，培养一批文艺人才、名家大师和文化领军人物。

增强文化产业竞争力。进一步完善文化产业发展规划，全力构建“一心两翼五基地十新城百大师”发展格局，将传统文化、艺术追求和现代时尚融入文化产业发展的全过程。促进文化与工艺美术产业融合发展，在品牌创建、技术创新、产业集聚上加大引导扶持力度，打造莆田工艺城、仙游博览城、上塘珠宝城百亿市场群。推进海西文化创意产业城建设，提升古典工艺家具、木雕、油画、金银珠宝首饰等业态，开发富有地域文化特色的工艺品。强化妈祖文化、南少林禅武文化、莆田工艺文化、“仙作”家具文化与旅游产业的融合，发展文化旅游产业。支持创意设计、演艺娱乐、体育竞赛、节庆会展、数字出版、影视动漫等新兴文化产业发展。

提高公共文化服务水平。建成市博物馆、图书馆、青少年宫，加快建设市科技馆，新建档案馆和30个文化激情广场，筹建市老年文化教育中心。抓好市、县区、乡镇、村四级公共文化设施建设，推动科教、文体、法律、卫生进农村、海岛、贫困地区。广泛开展群众性文体活动，丰富群众精神文化生活。加强新闻媒体建设和互联网监管，唱响主旋律，传播正能量。提高竞技体育水平，备战省运会，筹备青运会。开展丰富多彩的全民健身活动，办好全民健身运动会，增强全民健康体质。

（六）突出增进人民福祉

办好惠民实事。加大民生投入，着力办好东圳水库综合治理等16 件为民实事，进一步解决群众关心的饮水、就学、住房、交通、污水处理等问题。

优先发展教育。新建小区必须按标准配套建设小学和幼儿园，按人口比例配套建设初中学校。实施城区幼儿园和中小学扩容工程，新建10所公办幼儿园、7所中小学，新增4000个中小学学位。支持名校办分校、老校带新校、强校扶弱校，继续实施中小学“班班通”，推进“百校帮百校”、“教师校际交流”工作，实现普通高中学校全部达标，促进城乡教育均衡发展。做好进城务工人员随迁子女、留守孩子义务教育。提高职业教育质量，培养更多实用技能人才。落实加快高校发展26条措施，加快建设莆田学院、湄洲湾职业技术学院新校区。加强教师和学生德育工作。

提高卫生计生服务水平。续建市第一医院、市中医医院，筹建市肺科医院和市口腔医院，扩建市中心血站，新增医院床位800张。全面推行村卫生所标准化建设和药品零差率销售改革，减轻群众看病负担。稳步推进公立医院改革，完善市级医疗集团建设，支持和规范社会资本办医，抓紧建设新安国际医院，推进建设中国（莆田）质子治疗中心，发展壮大民营医院。建立市县（区）医院挂钩乡镇卫生院的帮扶机制，健全重大疾病医疗保险和救助制度，完善医疗急救、卫生应急管理体系。坚持计划生育基本国策，提高出生人口素质。

提供更有力社会保障。推动城乡居民社会养老保险并轨统一，养老保险金提高到每人每月70元，完善失地失海农民养老保障制度。实施城乡居民一体化医疗保险政策，实行基金市级统筹管理，人均年筹资标准提高到390元。有序推进棚户区、旧城区改造，开工建设1000套保障性住房和10348套安置房，继续抓好安置房历史遗留办证工作。整合社会救助资源，完善社会救助体系，发展慈善、社会福利和残疾人事业，农村低保月补助水平增加30元。加强敬老院、居家养老服务建设，鼓励社会力量参与养老事业，提高专业化养老服务水平。

扩大更高质量就业。加强城乡一体就业平台建设，促进高校毕业生、农村转移劳动力、城镇登记失业人员、退役军人就业，新增城镇就业2万人。加强职业技能培训，推进“名师带徒”工程，开展职业技能竞赛，建立产业技工培养基地。适时调整最低工资标准，严格劳动用工制度，依法维护企业和劳动者合法权益。

推进社会治理创新。培育发展行业协会、商会、科技、社区服务等社会组织，建立社会诚信评价、失信惩戒和“黑名单”等制度。创新多元调解机制，构建大调解格局，健全法律援助和社会救助制度，有效化解社会矛盾。健全重大决策社会稳定风险评估机制，完善联合接访制度，建立涉法涉诉信访依法终结制度，畅通群众诉求渠道，维护群众合法权益。落实安全生产责任，加强安全生产防范，继续开展道路交通安全综合整治和“清剿火患”专项行动，遏制各类事故发生。加强食品药品安全监管。深化“平安莆田” 建设，完善立体化社会治安防控体系，强化社区网格化管理，依法严密防范和惩治各类违法犯罪活动。完善防灾减灾体系，加强地震监测跟踪和科普宣传，提高自然灾害防御能力。支持工会、共青团、妇联等人民团体充分发挥桥梁纽带作用。认真落实党的民族政策和宗教工作基本方针。强化外事外侨工作，汇聚侨心侨智侨力。落实优抚政策，妥善安置转业和退役军人，支持驻莆部队、武警部队训练和后勤保障，深入推进爱民固边，加强民兵、预备役部队建设，增强海防、人防能力，推动军民融合深度发展。

（七）推进生态文明建设

加强环境治理与保护。持续开展创建国家生态市、国家森林城市、全国水生态文明城市和国家环保模范城市工作，创建5个省级生态县（区）、26个国家级生态乡镇。完善生态补偿和资源有偿使用制度，抓好木兰溪源省级自然保护区建设，保护东圳水库、外度水库等饮用水源。推进污水进管网，加快建设湄洲湾北岸尾水排放工程和北岸、仙游第二、闽中三期污水处理厂，铺设污水管网120公里，取缔直排河道的工业排污口。加大农村垃圾收集力度，推广生活垃圾分类，建设餐厨垃圾处理设施。严格河道（海域）采砂、矿产资源开采监管。深化畜禽养殖、环境卫生、大气污染、城市噪音和建筑运输扬尘等专项治理，开展PM2.5空气质量和内河水质监测工作。守住耕地红线，节约集约利用土地。实施“四绿”工程，加强沿海防护林基干林带、高速公路和中心城区环城一重山森林生态景观建设。

促进节能减排。严格执行环境影响评价制度，杜绝高排放、高污染、高耗能项目，鼓励引进节能环保型项目。引导企业制定能效对标方案，狠抓节电、节水、节材、节地和“三废”污染减排等，推进低氮燃烧技术改造和烟气脱硝工程建设，研究突破重点领域共性技术与关键技术。实施50个企业节能改造项目，促进重点耗能企业节能降耗，确保单位生产总值能耗下降3.3%以上。

发展循环经济。推广应用低碳技术，提高能源和资源综合利用率。推动莆田高新技术产业园、仙游循环经济示范园、秀屿临港工业园创建国家级循环经济园区。调整能源消费结构，发展清洁能源和可再生能源，扩大LNG天然气覆盖面与使用率。推行清洁生产，加强对重点耗能、耗水和污染企业清洁生产审核。建设再生资源回收体系，推进资源再生利用产业化。

三、全面加强政府自身建设

转变政府职能。把改革创新贯穿于经济社会发展的各个领域各个环节，推动政府职能向创造良好发展环境、提供优质公共服务、维护社会公平正义转变，促进资源要素市场化配置，充分激发市场主体活力，推广政府购买公共服务。完成政府机构改革，稳妥推进事业单位分类改革，加快推进各级党政机关与所办（属）企业及其管理的经营性资产脱钩改制，积极推进中介服务机构与行业主管部门脱钩。进一步做大做强八大集团，完善文化企业国有资产管理体制，加快建设国有企业现代企业制度，做到放权到位、监管到位。规范土地供应管理，推动土地资源依据市场规则、市场价格、市场竞争配置，继续清理供而未用土地，推动“三旧”改造，推行城市棚户区（危旧房）改造项目“毛地”出让方式，充分发挥土地经济效益。

优化发展环境。切实改进机关作风，加大效能问责力度，激发干事创业热情。本利莆利民之心、行利莆利民之政，勇于担当、敢于承担，到基层、到一线发现问题、协调问题、解决问题，为群众办好事，让群众好办事。研究政策、制定政策、用好政策，释放更多的改革红利惠民惠企。深化行政审批制度改革，再造流程，能减则减、能并则并、能放则放、能优则优，大力推行并联审批、容缺预审，推进行政审批服务标准化。进一步规范招投标等制度，凡能用备案的不搞审批，凡能事中事后监管的不搞事前前置，变事前审批为事中、事后监督与服务，推动更多环节从审批转为备案，促进项目加快上马。按照“非禁即入”原则，制定市场准入负面清单，推进工商注册便利化。

坚持依法行政。自觉接受市人大及其常委会的法律监督、工作监督和政协的民主监督，强化层级和专门监督，高度重视社会和舆论监督。加强行政协商，完善科学民主依法决策机制，建立决策后评估纠错制度，提高政府决策水平。加强行政复议和应诉工作，不断提高依法行政水平。认真落实行政执法责任制和执法过错追究制，坚持文明执法。

严格廉洁从政。扎实开展党的群众路线教育实践活动和领导干部“四下基层”活动，贯彻落实中央“八项规定”，坚决纠正“四风”。厉行节约、反对浪费，加强“三公”经费管理，停止新建楼堂馆所，规范公务接待管理，严格控制会议经费和庆典、节会、论坛等活动，市本级预算单位公用经费压缩5%以上。加强行政监察和审计监督，加大政务公开力度。健全惩治和预防腐败体系，坚决纠正损害群众利益的不正之风，保证人民赋予的权力始终用来为人民谋利益，努力实现干部清正、政府清廉、政治清明。

各位代表，加快建设“人兴业茂、清风明月”的宜居港城，是全市人民的共同愿景，也是时代赋予我们的光荣使命。让我们在中共莆田市委的坚强领导下，凝聚300多万莆田人民的磅礴力量和无穷智慧，同心同德，改革创新，锐意进取，攻坚克难，在壶山兰水这片充满生机活力的热土上，奋力谱写“美丽莆田、幸福家园”的崭新篇章！
